# Development of a Patch-Type Flexible Oxygen Partial Pressure Sensor

**DOI:** 10.1109/JTEHM.2020.3005477

**Published:** 2020-06-29

**Authors:** Yuta Katayama, Yuta Fujioka, Kosuke Tsukada

**Affiliations:** 1 Graduate School of Fundamental Science and TechnologyKeio University 12869 Yokohama223-8522Japan; 2 Department of Applied Physics and Physico-InformaticsKeio University 12869 Yokohama223-8522Japan

**Keywords:** Oxygen sensor, phosphorescence, wearable, point-of-care testing

## Abstract

Oxygen concentration in living organisms is one of the important vital indicators in emergency care and bedside medical settings. However, the oximetry method has limitations: the measurement site is limited to the tissue containing blood and the absolute value of oxygen concentration cannot be measured. To overcome these limitations, in this work, we develop a new oxygen sensor that can directly measure the oxygen particle pressure ($p\text{O}_{2}$) on the surface of the body and organs. A light emitting diode (LED) and a photodiode (PD) were embedded in a dimethylpolysiloxane substrate mixed with carbon nanotubes. The effectiveness of the device was evaluated using calibration, bending strain tests, time and frequency response, and finally *in vivo* assessments. The results reveal that the calibration experiment of the fabricated oxygen sensor device showed high sensitivity. The carbon nanotube electrode has a sufficient bending resistance and does not affect the response characteristics of the LED and PD, that is, it does not affect the oxygen measurement. *In vivo* assessment shows that the developed patch-type flexible oxygen sensor can accurately measure $p\text{O}_{2}$ by attaching it to tissues or organs having irregularities or curved surfaces and actual measurements on rat liver surface demonstrated its feasibility.

## Graphical abstract

We present a new oxygen sensor that can directly measure the oxygen particle pressure (pO2) on the surface of the body and organs. In vivo assessment shows that the developed patch-type flexible oxygen sensor can accurately measure pO2 with high sensitivity on tissues or organs with irregularities or curved surfaces.

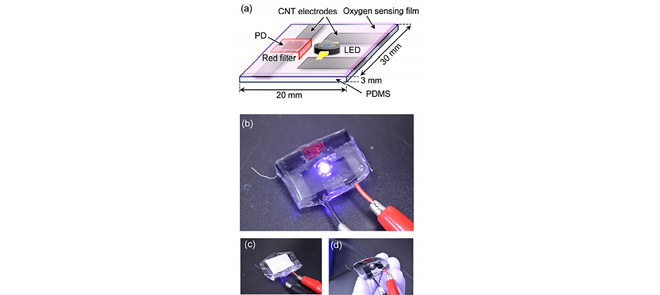

## I. Introduction

In the medical field, oxygen concentration is one of the vital signs from living organisms, especially in bedside and emergency medical settings. Moreover, in experiments and research using animals and cultured cells, the oxygen concentration in living organisms is often measured. A report has shown that oxygen concentration control in a culture environment is important in regenerative medicine where oxygen concentration determines cell differentiation [1].

Presently, pulse oximetry using the absorbance of hemoglobin is commonly used in clinical practice [2], [3], and the oxygen electrode method has been generally used at the laboratory level [4]–[6]. Pulse oximetry has the advantage of non-invasive measurement, which has found widespread application for humans. However, it has some disadvantages; for example, the measurement site is limited to tissues containing sufficient blood and the absolute value of oxygen concentration cannot be measured. Alternatively, the oxygen electrode method has the advantage of absolute value measurement, but is limited to measurement in liquid containing electrolyte, and involves physical invasion such as insertion of a sensor. The measurement of oxygen partial pressure ($p\text{O}_{2}$) by the phosphorescence lifetime method [7], [8], which is based on the oxygen quenching effect of phosphorescence (where $p\text{O}_{2}$ is calculated from the intensity or lifetime of the luminescence emitted depending on oxygen), has been applied to various organs [9]–[12]. It is relatively minimally invasive because the excitation light is applied to the site where the phosphorescence dye exists. In addition, it has a high gas selectivity because the oxygen molecule is an unusual gaseous molecule, usually in the triplet ground state, and only the oxygen molecule can receive energy from dye molecules in the excited triplet state [13], [14]. However, it is limited to animal use and it is difficult to administer the dye to humans because of its phototoxicity of its energized singlet oxygen. [15], [16].

Local oxygen concentration measurement has been enabled by embedding a photosensitive dye in a polymer to form a film and attaching it to the measurement site. For example, platinum octa-ethyl porphyrin (PtOEP) was directly incorporated into IMPEK-C polymer to form a film, which enabled high-precision oxygen measurement using light emitting diodes (LEDs) and photodiodes [17]. Furthermore, measurement sensitivity was improved by incorporating PtOEP into a polystyrene (PS) polymer matrix and forming it into a porous-structured oxygen-sensing film [18]. Recently, our group has developed PS particles containing oxygen-sensitive dyes and a flexible oxygen-sensing film containing them in dimethylpolysiloxane (PDMS) and applied it to the quantification of respiratory activity of cultured cells [19]. The advantages of the sensor film are that the dyes do not contact the living body directly and can be applied in both gas and liquid. These properties enable safe application to living organisms and are expected to be applied to point-of-care testing (POCT). However, these film-type oxygen sensors require a light source for photoexcitation and a detector to detect luminescence and the measurement equipment tends to be large. Therefore, it is necessary to integrate the optical components and sensor film and configure the wiring with a flexible material to apply it to POCT.

In this study, the LED for exciting the oxygen-sensitive dye and the PD for phosphorescence detection were integrated with the oxygen sensor film. Moreover, a flexible oxygen sensor based on the phosphorescence lifetime method was developed by connecting the optical elements through carbon nanotubes (CNTs). Calibration, bending strain tests, and time and frequency response were used to validate the device as a sensor, and actual measurements on rat liver demonstrated its feasibility.

## II. Materials and Methods

### A. Sensor Design and Fabrication

**FIGURE 1.**
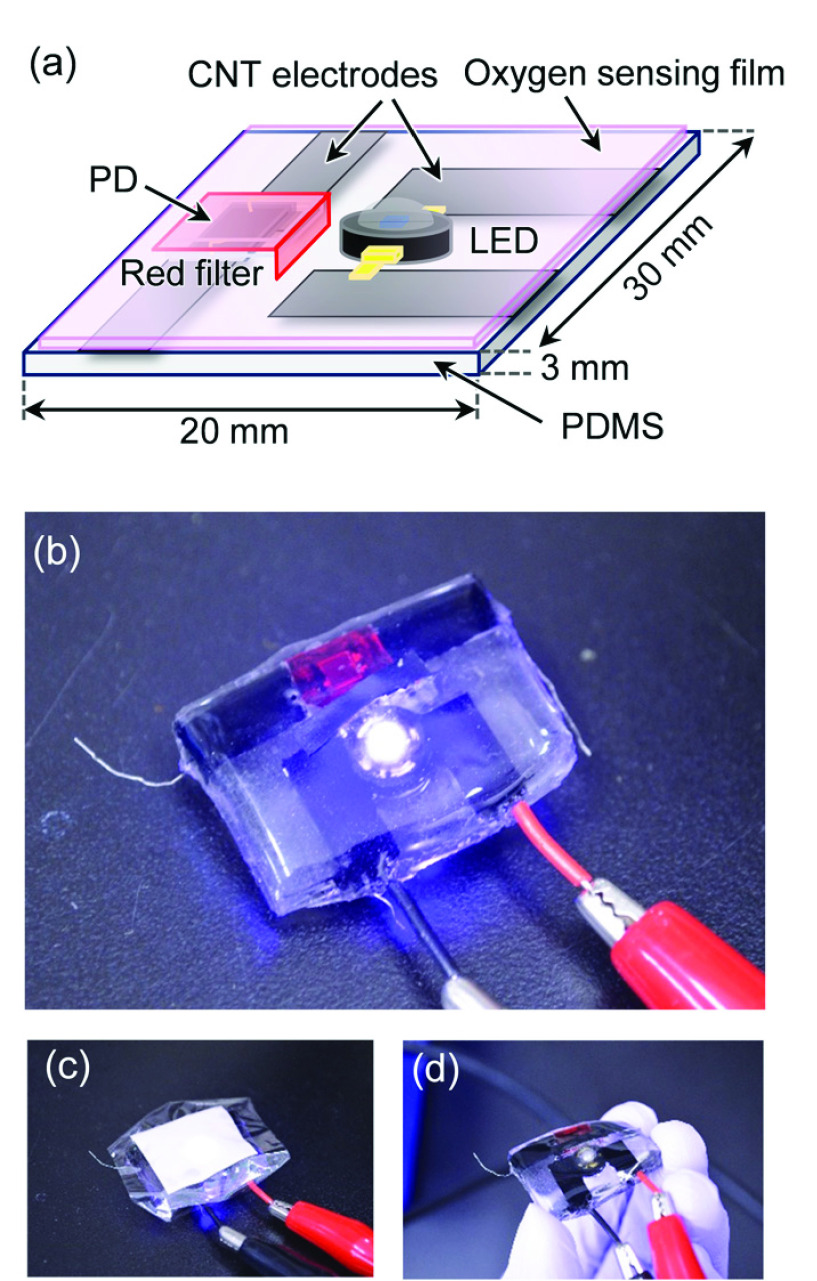
(a) Schematic of the oxygen sensor device and photographs of (b) the entire sensor, (c) with oxygen sensor film and (d) bent state.

A schematic and a photograph of a patch-type oxygen sensor in which the light source, detector, and sensing film are integrated are shown in Figure 1(a, b). PDMS was used as the base material of the sensor. An LED (PM2L-1LLE-LC, Pro-Light) with a peak wavelength of 395 nm and a PD (VBPW34S, VISHAY) were used as the excitation light source and detector, respectively; these were embedded in a PDMS substrate. An oxygen sensor film (PDMS containing oxygen-sensitive polymer particles) [19] was bonded to the device facing the LED and PD, as shown in Fig. 1(c), and the film was attached to the surface of the measurement target. The flexible characteristics of PDMS allow the sensor device to bend, as shown in Fig. 1(d).

The LED and PD devices were both connected through a mixture of PDMS and carbon nanotubes (CNTs/PDMS) as previously reported [20]. Briefly, 120 mg of multi-walled CNTs (multi-walled, >98% carbon basis, O.D. $\times $ L 6-13 nm $\times2.5$-$20~\mu \text{m}$, Sigma-Aldrich) were mixed with 6 g of 2-isopropyl alcohol (IPA) and sonicated for 30 min for dispersion. Subsequently, 0.20 g of PDMS with a viscosity of 100 cSt was added and the resulting mixture was sonicated again for 30 min. Then, 0.80 g of PDMS-A (SILPOT 184 Base, Dow Corning Toray) was added and the resulting mixture was sonicated for another 30 min. This mixture was subjected to heating with a heater for two weeks at 55 °C to completely remove IPA. After the heating process, 0.10 g of polymerized material PDMS-B (SILPOT 184 Agent, Dow Corning Toray) was added and thoroughly mixed. The defoamed mixture was poured onto the PDMS substrate to form wires. The 12-wt% CNTs/PDMS prepared by the above procedure was used for LED wiring. Moreover, the wiring on the PD side, where the distortion is greater than that on the LED side, was achieved with an 8-wt% mixture prepared by the same procedure.

**FIGURE 2.**
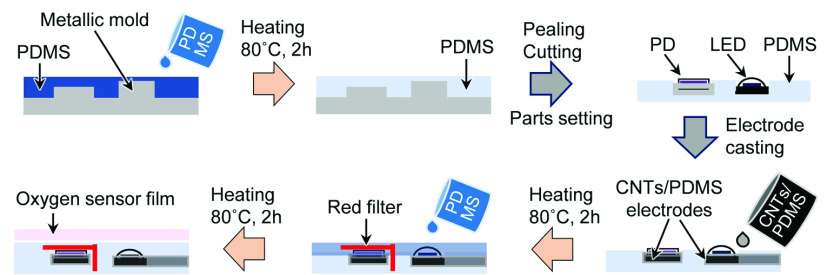
Preparation procedure for the sensor device.

Figure 2 shows the fabrication procedure of the sensor device. The PDMS substrate was formed by mixing PDMS-A and PDMS-B in a ratio of 10:1 vol% and then degassed by evacuation. The mixture was poured into a designed metal mold, and finally heated at 80 °C for 2 h. The LED and PD were placed on the PDMS substrate, and the CNTs/PDMS was cast on the connection and heated at 80 °C for 2 h for curing. A long-pass filter with a cut-on of 600 nm was installed on top of the PD, and to cover the CNTs/PDMS wire surface, a thin coating of PDMS was applied. The size of the oxygen-sensitive PS particles in the film was $1.3~\mu \text{m}$ and the film thickness was 200 $\mu \text{m}$. The oxygen-sensing film was bonded on the device before measurement. The detailed method for preparing the oxygen-sensing film has been described in a previous paper [19].

### B. Calibration of pO_2_ and Response Time Experiment

The sensor device was set in a gas-tight chamber, and the $p\text{O}_{2}$ inside was set from 0 to 159 mmHg every 40 mmHg by changing the mixing ratio of nitrogen gas and compressed air. After the $p\text{O}_{2}$ in the chamber was stabilized, a voltage (peak-to-peak voltage 10 V and offset voltage 5 V, frequency 120 Hz, rectangular wave, and duty ratio 1:4) was applied to the LED and the phosphorescence emitted from the oxygen-sensing film was detected by the PD. The current from the PD was converted into a voltage using a trans-impedance amp (T-Z amp), and a phosphorescence decay curve was obtained at a sampling frequency of 1 MHz and 6140 sampling points using a DAQ (USB 6363, NI instruments). The lifetime was calculated only within the range of 10%–50% of the intensity of the luminescence decay to eliminate light leakage and switching noise from the LED. Furthermore, $p\text{O}_{2}$ was quantified from the Stern–Volmer equation [21].

\begin{equation*} \tau _{0} / \tau =1+k_{\mathrm {q}} \tau _{0} [pO_{2}] = 1+K_{\mathrm {SV}} [pO_{2}]\tag{1}\end{equation*}

where $\tau _{0}$ and $\tau $ are the phosphorescence lifetimes at 0 mmHg and [$p\text{O}_{2}$], respectively, and $k_{\mathrm {q}}$ and $K_{\mathrm {SV}}$ are quenching rate and Stern–Volmer constants, respectively.

To determine the response time of the oxygen sensor, the $p\text{O}_{2}$ in the gas chamber was changed stepwise at 0 mmHg and 159 mmHg every 15 min, and the time required to reach 95% of the target $p\text{O}_{2}$ was evaluated as the response time [17], [21].

### C. Electrical Properties of CNTs/PDMS Wires Against Bending Strain

To measure resistivity (equal to the reciprocal of conductivity) of CNTS/PDMS, copper tapes were placed on both ends of a 50 mm $\times25$ mm PDMS substrate and fixed with conductive paste (SKU-0018, Bare Conductive). A rectangular (5 mm $\times3$ mm $\times25$ mm) CNTs/PDMS wire was fabricated on the PDMS substrate. Plastic plates were also placed at both ends of the PDMS substrate, and one side was fixed to a table and the other was fixed to a moving stage. Bending strain was applied to the CNTs/PDMS wire by moving the stage by 2 mm, and the bending angle measured at each position. The resistivity was then calculated by applying a current of 1 mA to the wire.

### D. Frequency and Response Characteristics to Bending Strain

To apply bending strain, the same procedure used for resistivity measurement was applied. At each bending angle, a sine wave (peak-to-peak voltage 2 V and offset voltage 4 V) was applied to a red LED (L-53HD, Kingbright) installed above the PD, and the frequency was swept from 10 Hz to 1 MHz. The output current of the PD was converted into a voltage $V_{\mathrm {pp}}$ and normalized by the output voltage ($V_{\mathrm {f}} =10$ V) at a frequency of 10 Hz. A rectangular current of 100 Hz was applied to the LED at each bending angle, and the voltage output ($V_{\mathrm {out}}$) from the PD was measured to obtain the response time of the PD.

### E. PO_2_ Measurement on Rat Liver Surface

Seven-week-old male Sprague Dawley rats were used for the experiment. These rats were placed on a heater pad maintained at 37°C, and anesthesia was induced with 2% isoflurane through an anesthesia mask and then maintained at 1.2%. A midline incision was made in the abdomen, followed by a transverse incision to expose the liver. An oxygen-sensing film was attached to the left lateral lobe of the liver and was covered with plastic wrap to prevent the atmospheric air from entering, and the sensor device was attached from above. The concentration of oxygen flowing into the anesthesia mask was reduced from 159 to 80 mmHg for 2 min by mixing equal amounts of nitrogen gas and air and then returned to 159 mmHg. The $p\text{O}_{2}$ on the liver surface was continuously measured by an oxygen sensor, and simultaneously, the $p\text{O}_{2}$ of the anesthetic gas was monitored with an oximeter (JKO-25, JIKCO).

## III. Results and Discussion

### A. PO_2_ Calibration and Response Experiments

**FIGURE 3.**
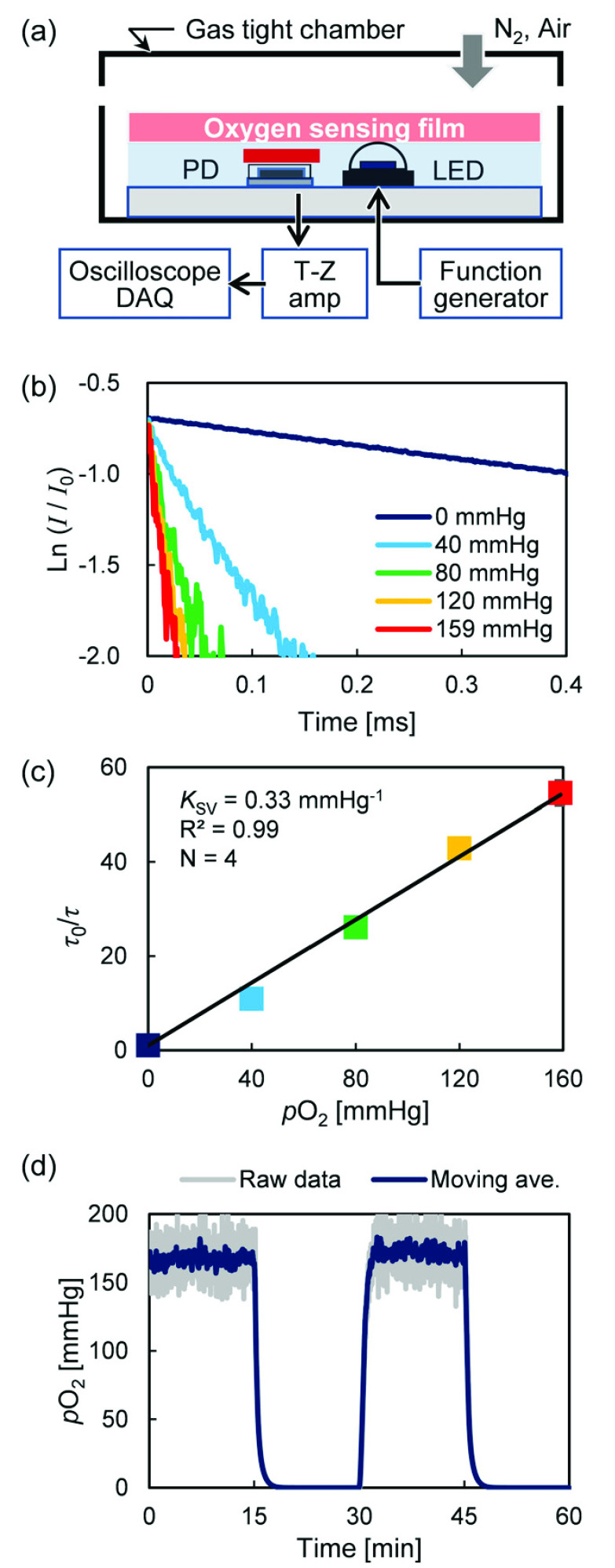
(a) Sensor installed in gas-tight chamber for calibration, (b) oxygen-dependent phosphorescent decay, (c) Stern–Volmer plot, and (d) response time results.

Figure 3(a) shows an oxygen sensor that was installed in the gas-tight chamber, and the oxygen concentration inside the chamber was changed stepwise from 0 to 159 mmHg in steps of 40 mmHg. Figure 3(b) shows the phosphorescence decay waveform for each oxygen concentration. Consequently, in the oxygen measurement method that is based on the oxygen quenching effect of phosphorescence in principle, the quenching by oxygen molecules reduces the phosphorescence intensity and shortens the lifetime; this leads to decrease in accuracy as the oxygen concentration increases, At 120 and 159 mmHg, the lifetime intensity change of the decay is small and noisy. However, the $p\text{O}_{2}$ of a living body is generally less than 100 mmHg, and moderate averaging and least-squares fitting can improve its accuracy. Furthermore, Fig. 3(c) shows a Stern–Volmer plot obtained from the phosphorescence lifetime, where $K_{\mathrm {SV}} =0.33$ mmHg^−1^, R^2^ = 0.99, indicating that this sensor is capable of high-sensitivity and high-accuracy oxygen sensing in the biological measurement range.

The response characteristics of the sensor when the $p\text{O}_{2}$ of the gas-tight chamber was changed stepwise every 15 min at 0 and 159 mmHg is shown in Fig. 3(d). The time to reach 95% of the target $p\text{O}_{2}$ was 95 s from 159 down to 0 mmHg and 83 s from 0 to 159 mmHg. The time delay caused by the sensor device is approximately 30 s because the reaction time of the oxygen-sensing film is approximately 57 s [19]. This shows that the response time is mainly determined by the oxygen-sensing film, and increasing the response of the film improves the response of the oxygen sensor. However, this response time includes the time delay caused by the experimental system because it took a certain time to replace the gas in the chamber.

Generally, a $p\text{O}_{2}$ change every second as a biological response under physiological conditions is rare. Therefore, the response speed of this device is sufficient for constant oxygen monitoring of patients and application to oxygen monitoring of cultured cells. This application of oxygen sensors to POCT technology requires further reduction in response time. This is because when applied to the skin and organs in emergency care, it must respond to sudden changes in oxygen. Moreover, if it is used for intraoperative oxygen monitoring, such as cardiac ischemia/reperfusion, a response time in seconds is required. This observation shows that the main factors that determine the response time of the oxygen-sensing film are the thickness of the PDMS and the particle size of the PS beads containing the oxygen-sensitive probe. Further improvements in the future will facilitate faster response time.

### B. Electric and Frequency Characteristics Under Bending

**FIGURE 4.**
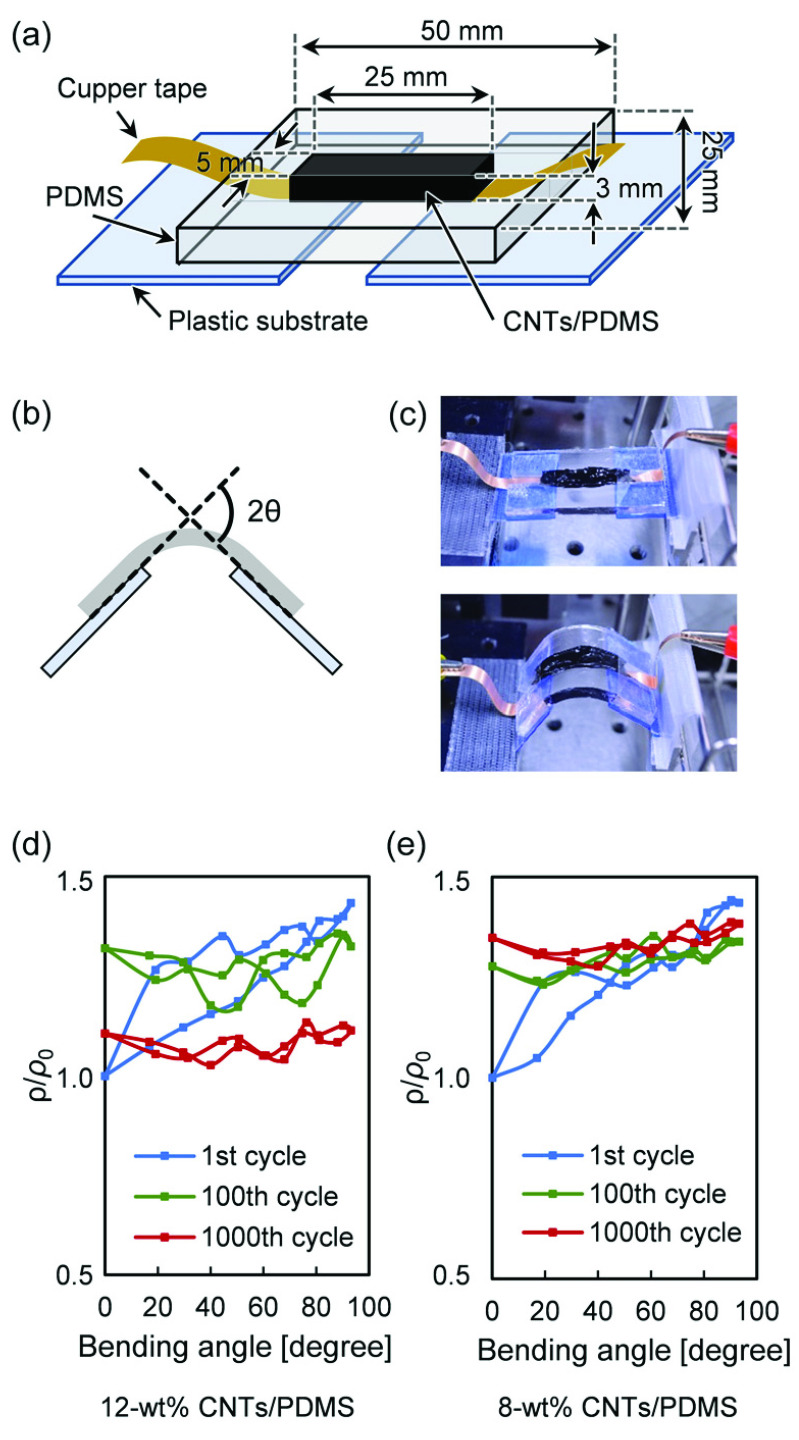
(a) Schematic of bending resistance test equipment for CNTs/PDMS wires, (b) measurement angle and (c, d) photographs of test condition and (e) angle-dependent resistivity change in 12 and 8-wt% wires.

The feature of the proposed oxygen sensor device is that optical elements are installed on a soft PDMS substrate and wired using PDMS mixed with carbon nanotubes so that it can be attached to the surface of a living body with complicated curvature. Therefore, electrical and frequency characterizations against bending need to be conducted. A bending strain was applied to the CNTs/PDMS wire with the same width (5 mm) and height (3 mm) as that in the connection with the oxygen sensor device as seen in (Fig. 4(a–c)). Figures 4(d, e) show the resistivity depending on the bending angle of 12-wt% and 8-wt% CNTs/PDMS used for LED light emission and PD light-receiving circuit, respectively. In the first strain, the angle-dependent resistivity increased as the angle increased, the repeated strain reduced the change, and the resistivity became stable at the 1000th cycle. This is because repeated application of strain stabilizes CNTs in the wires [20], [23], [24]. The resistivity increased with the number of strains, but it was approximately 1.4 times at the maximum, which shows that the actual change in resistance was as small as 287–$386~\Omega $ in 8-wt% CNTs/PDMS. Similar characteristics of 12-wt% CNTs/PDMS suggest that the distortion does not affect the operation of the LED and PD.

**FIGURE 5.**
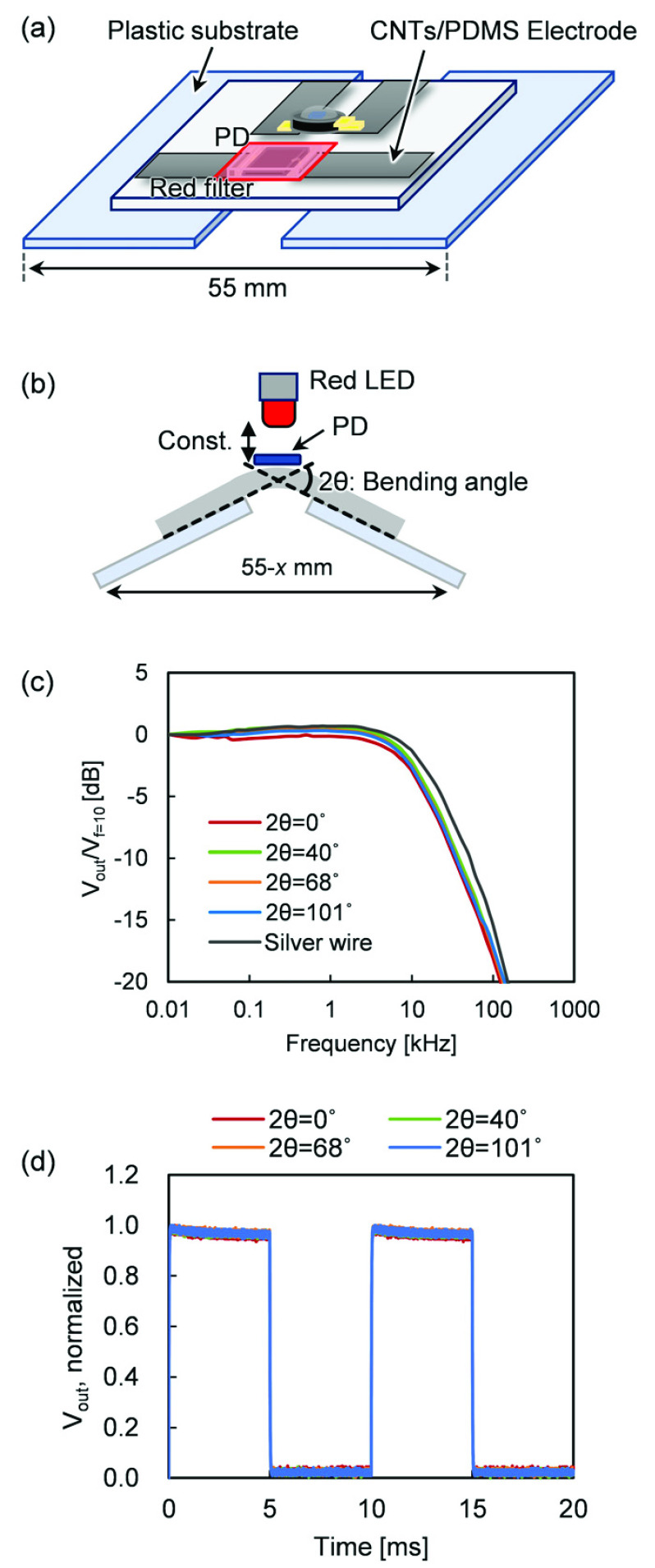
(a) Schematic of equipment for bending resistance test of optical elements, (b) arrangement of optical elements and bending angle, (c) frequency characteristics of PD output, and (d) response time test result.

Even though the increase in the resistivity of the CNTs/PDMS reduces the LED and PD currents, the signal intensity of the phosphorescence decreases, while the lifetime remains constant. Thus, this method of quantifying $p\text{O}_{2}$ from the lifetime enables robust measurement. Furthermore, to clarify the influence of the sensor device distortion on the life measurement, the frequency characteristics for the distortion were obtained. Strain was applied while changing the bending angle of the sensor device, and a sinusoidal current from 10 Hz to 1 MHz was applied to the LED (Fig. 5(a, b)). Figure 5(c) shows that the frequency response of the PD output does not depend on the distortion angle. In other words, the time difference between the LED and PD mainly depends on the response time of the PD, which is constant regardless of the distortion angle of the sensor device. Basically, the output response of the PD when the LED is driven rectangularly at 5 ms intervals does not depend on the distortion angle (Fig. 5(d)). Furthermore, the result was equivalent to that for silver wires (black solid line). From these two strain experiments, it can be concluded that the CNTs/PDMS wire is suitable for phosphorescence lifetime-based $p\text{O}_{2}$ measurement. However, at the contact point between CNTs/PDMS and the sensor electrode, a change in resistance or contact failure may occur due to the difference in rigidity between the PDMS and the sensor device, that is, mechanical mismatch, which reduces the signal-to-noise ratio. As mentioned above, this sensor, which calculates $p\text{O}_{2}$ from phosphorescence lifetime rather than intensity, is resistant to noise. In addition, as shown in Fig. 5 (c), bending stress did not affect PD output and response time even at an angle of 100 degrees. However, it is necessary to fully consider the mechanical mismatch when changing the thickness of PDMS and the size of the device in order to further miniaturize the sensor and improve its flexibility.

### C. PO_2_ Measurement on Rat Liver Surface

**FIGURE 6.**
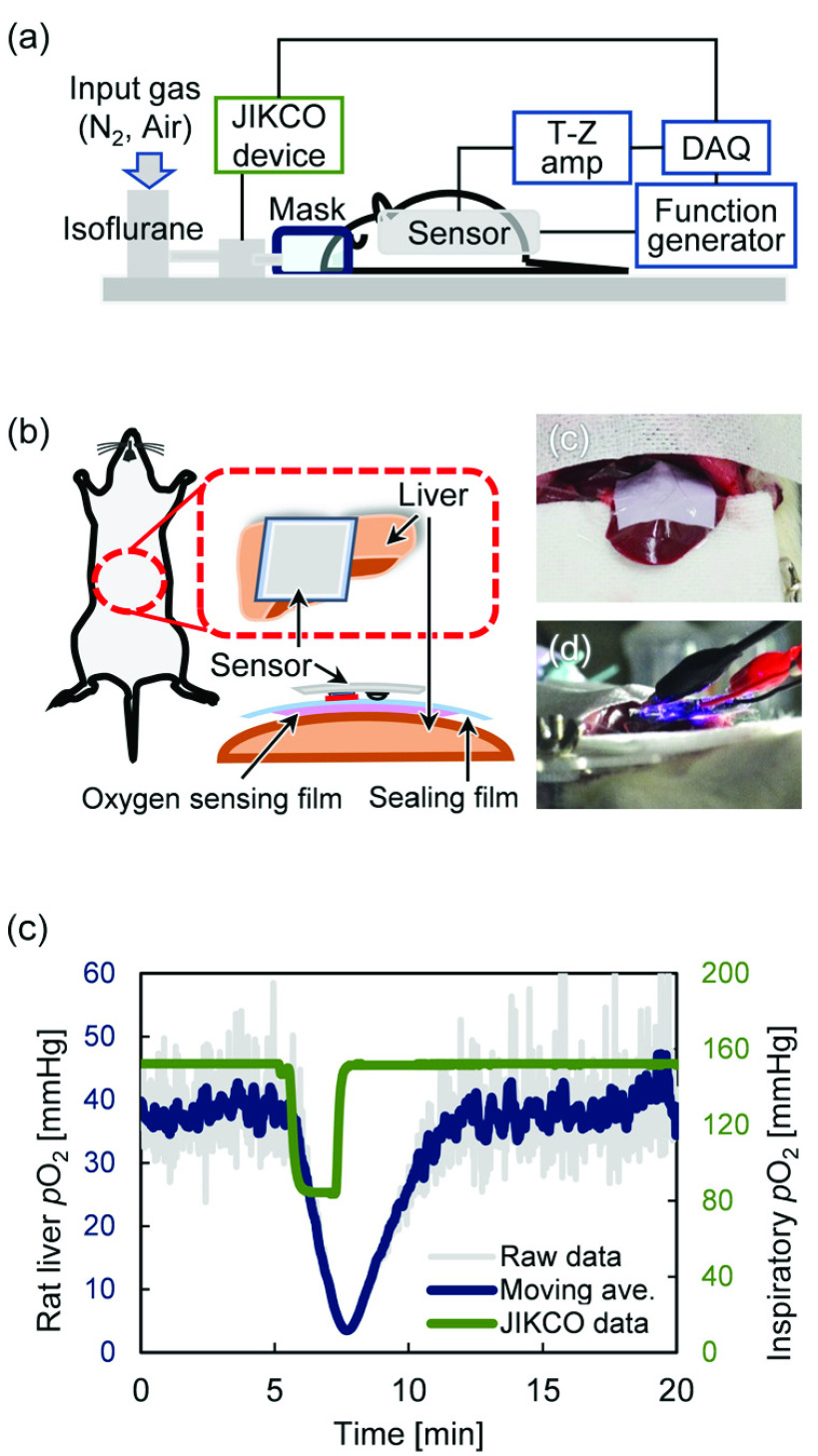
(a) Schematic of animal experiment, (b–d) outline and photographs of attachment of the sensor device to rat liver, and (e) $p\text{O}_{2}$ change on liver surface with changes in inspired oxygen concentration.

To evaluate the effectiveness of the developed oxygen sensor device, $p\text{O}_{2}$ measurement was performed on the rat liver surface. Gas containing isoflurane was supplied to rats through an anesthesia mask, and the oxygen concentration was reduced to 80 mmHg for 2 min (Fig 6(a)). Liver was selected as the subject because of its large size, easy access after laparotomy, and expected large changes in $p\text{O}_{2}$. First, as shown in Fig. 6(b–d), the oxygen sensor film was attached to the liver surface, and then a wrap was placed on it to shut off the outside air, and the sensor device was placed on it. The $p\text{O}_{2}$ on the liver surface was maintained at approximately 40 mmHg until the oxygen in the inhaled gas reduced (Fig. 6(e)). Much of the blood supplied to the liver comes from the portal vein. The $p\text{O}_{2}$ of the peripheral branch of the portal vein, which merges with blood derived from the hepatic artery, is approximately 70 mmHg, while that of the central vein is approximately 40 mmHg [25]. Therefore, $p\text{O}_{2}$ of 40 mmHg before hypoxia stimulation is a physiologically stable value. Shortly after the $p\text{O}_{2}$ in the inhaled gas was reduced by half, the $p\text{O}_{2}$ on the liver surface began to decrease, fell to a minimum of 4 mmHg, and recovered over time. This time lag between the change of inhaled gas and liver $p\text{O}_{2}$ was considered to be caused by the volume of gas in the inhalation mask, gas exchange in the lungs, and systemic hemodynamics. The result shows that the recovery was slower than the decrease in $p\text{O}_{2}$; this may be because of the main blood flow into the liver that comes from the portal vein. Also, the raw data showed small fluctuations of approximately ±10 mmHg owing to the effects of breathing and extraneous noises, while the moving average processing could stabilize the signal. Similar experiments were performed on the reproducibility of the same individual and on a separate body (Supplemental figures). Although similar differences were observed depending on the depth of anesthesia and the site where the sensor was attached, similar changes in $p\text{O}_{2}$ were observed, indicating that the oxygen sensor device enables repeated and stable measurements. In addition, the sensor must sufficiently block ambient light during measurement. Liver $p\text{O}_{2}$ was measured in a dark room, but when this sensor is used for POCT under daylight, stray light passing through the red filter and reaching the sensor becomes background noise, which reduces measurement accuracy. As mentioned above, $p\text{O}_{2}$ is calculated from the lifetime rather than the phosphorescence intensity, so, theoretically, DC noise does not affect the measurement accuracy, but partial shading of the PDMS substrate may contribute to accuracy improvement.

Currently, pulse oximetry, which is used in clinical practice, is a method that uses the difference in absorbance of redox hemoglobin in near-infrared light. The simple and non-invasive measurement method enables a wide range of applications from emergency to bedside monitoring. However, it involves a redox state of hemoglobin in arterial blood contained in irradiated tissue, that is, it has a systemic oxygen concentration index, which serves as a drawback to its function. On the other hand, this newly developed oxygen sensor can directly measure the absolute oxygen concentration of the “local parenchyma” and can be applied to relatively low blood vessel density, such as in skin tissue. However, the phosphorescence lifetime is temperature-dependent, and it is necessary to apply a suitable $K_{\mathrm {SV}}$ for tissue temperature to quantify the $p\text{O}_{2}$, or to add a temperature compensation function to oxygen-sensing films or optical devices.

In the future, further thinning of the sensor device is expected to enhance its flexibility and make it applicable to a moving heart or the brain surface with complex irregularities. By arranging optical elements at high density, two-dimensional $p\text{O}_{2}$ imaging will be possible in the future. It is expected to develop into an oxygen imaging device that can visualize local hypoxic regions of the heart and brain and the changes during ischemia/reperfusion. In addition, the patch type 2D oxygen sensor will be applied to POCT; e.g., for visualization of $p\text{O}_{2}$ distribution in the heart, lungs, and liver during surgery, percutaneous $p\text{O}_{2}$ measurement independent of CO hemoglobin in emergency medicine, and for use as a bedside exhalation gas monitor. Furthermore, mounting of a sensor on the endoscope will be developed into a method for detecting a tumor site exhibiting hypoxia.

## IV. Conclusion

A patch-type oxygen sensor composed of a flexible material has successfully been developed by our group, and it has been demonstrated that it can be applied to curved tissues and organs to measure $p\text{O}_{2}$ accurately. The carbon nanotube electrode has a sufficient bending resistance of at least 1000 times and does not affect the response characteristics of the LED and PD; that is, does not affect the oxygen measurement. In vivo assessment shows that $p\text{O}_{2}$ can be accurately measured by attaching the developed patch-type flexible oxygen sensor to tissues or organs having irregularities or curved surfaces, and the actual measurements on rat liver surface demonstrated its feasibility. This $p\text{O}_{2}$ measurement on the rat liver surface proved that the sensor is useful for biological measurement. These results indicate that this sensor device helps to mitigate the challenge of limited measurement site problem associated with hemoglobin-based pulse oximetry. This newly developed sensor is expected to evolve into an oxygen imaging device by further thinning and increasing the number of optical elements and to be applied to emergency use and bedside point-of-care tests.
